# Supplementary material for: Characterization of a New Temperate Escherichia coli Phage vB_EcoP_ZX5 and Its Regulatory Protein
Source: Pathogens. 2022 Nov 30;11(12):1445. doi: 10.3390/pathogens11121445 (PMC9782041; doi:10.3390/pathogens11121445)
Supplement: Supplementary file 1 [file pathogens-11-01445-s001.zip › pathogens-1997316-supplementary.pdf]

Supplementary Materials

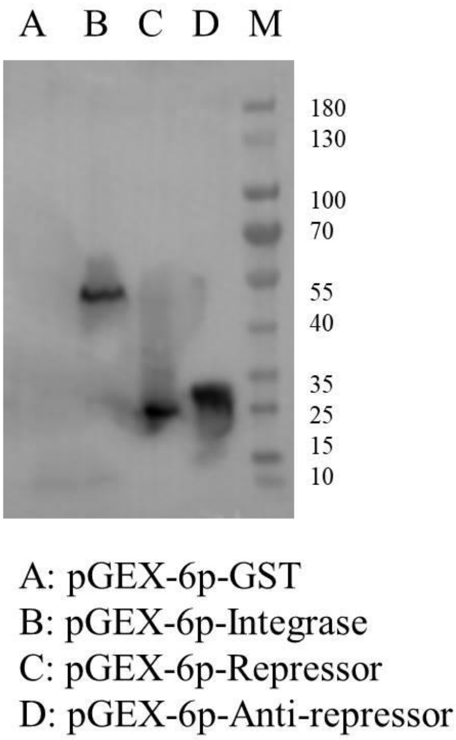

Figure S1: Western blot of protein expression. A: *E. coli* YO1 carrying GST tag overexpression plasmid was induced by IPTG (negative control); B: *E. coli* YO1 carrying integrase overexpression plasmid was induced by IPTG; C: *E. coli* YO1 carrying repressor overexpression plasmid was induced by IPTG; D: *E. coli* YO1 carrying anti-repressor overexpression plasmid was induced by IPTG.

Table S1. Primers used in this study

**Primers**	**Sequence**	**Tm (°C)**	**Size (bp)**	**Target gene/ORF**
fliCF	ATGGCACAAGTCATTAATACCCAAC	54	1497	*fliC* in *E. coli* YO1
fliCR	CTAACCCTGCAGCAGAGACA	53		
K1F	CATCCAGACGATAAGCATGAGCA	55	250	*kpsM* in *E. coli* YO1
K1R	GCGCATTTGCTGATACTGTTG	52		
aatAF	CTGGCGAAAGACTGTATCAT	50	630	*aatA* in EAEC
aatAR	CAATGTATAGAAATCCGCTGTT	50		
aggRF	CTAATTGTACAATCGATGTA	45	308	*aggR* in EAEC

aggRR	ATGAAGTAATTCTTGAAT	43		
eaeF	GACCCGGCACAAGCATAAGC	56	384	*eae* in STEC and EPEC
eaeR	CCACCTGCAGCAACAAGAGG	56		
bfpAF	CAATGGTGCTTGCGCTTGCT	54	324	*bfpA* in EPEC
bfpAR	GCCGCTTTATCCAACCTGGT	54		
ltAF	GGCGACAGATTATACCGTGC	54	450	*ltA* in ETEC
ltAR	CGGTCTCTATATTCCCTGTT	50		
stAF	ATTTTTMTTTCTGTATTRTCTT	46	190	*stA* in ETEC
stAR	CACCCGGTACARGCAGGATT	53		
ipaHF	CTCGGCACGTTTTAATAGTCTGG	55	917	*ipaH* in EIEC
ipaHR	GTGGAGAGCTGAAGTTTCTCTGC	57		
stx1F	ATAAATCGCCATTCGTTGACTAC	52	188	*stx1* in STEC
stx1R	AGAACGCCCACTGAGATCATC	55		
stx2F	GGCACTGTCTGAAACTGCTCC	56	255	*stx2* in STEC
stx2R	TCGCCAGTTATCTGACATTCTG	53		
1F	GAATTGTGCTTACCGATAC	49	1246	ORF12 integrase
1R	CCATCCACTTCTCAAACTTCG	52		
2F	GTGACAGATACAGCGTTA	48	1473	ORF34 terminase large subunit
2R	GCATAGGGATCGTAATCTGT	50		
3F	TGCCGTACGATTTCCTCGGT	54	286	attB in *E. coli* YO1
3R	ATGACTGAAGGCGGTCGCGATCTG	58		
3F2	TGGTACTGATTTCCGGTGACGGT	57	281 (3F2/3R)	attR in *E. coli* YO1+

3R2	TATGGTGTAGAGTGAATAG	47	214 (3F/3R2)	attL in *E. coli* YO1+
4F	TTGTAAGGTCGTGGTGACCG	56	203	ORF34 terminase large subunit in phage vB_EcoP_ZX5
4R	CGAATGCCTCAGTGTTGTGC	56		
5F	CAGGAAACAGTATTCATGTCCATGACAGCTACAAAAAGC	64	771	ORF4 anti-repressor in phage vB_EcoP_ZX5
5R	TTAGTGATGATGGTGGTGATGGAAGAACCCTGCTTTGTC	66		
6F	CAGGAAACAGTATTCATGTCCGTGCTTACCGATACAAAA	64	1326	ORF12 integrase in phage vB_EcoP_ZX5
6R	TTAGTGATGATGGTGGTGATGTTCCCCCGCCATCCACTT	67		
7F	CAGGAAACAGTATTCATGTCCATGAAAAGTATCCAGGAC		660	ORF21 repressor in phage vB_EcoP_ZX5
7R	TTAGTGATGATGGTGGTGATGTTGTTTGTTTTTTTCGTT	64		
8F	TCGGAAGCTGTGGTATGGCTGTGCA	61	1000	plasmids pGEX-6P-1
8R	CCGGGAGCTGCATGTGTCAGAGG	62		
9F	GAGCGAACACTGACACCAGA	58	200	ORF12 integrase in phage vB_EcoP_ZX5
9R	CTTCCCTTCATGCGGTCTGA	58		
10F	AATGGTGTTCAGACGCGGAT	56	171	ORF21 repressor in phage vB_EcoP_ZX5
10R	ACCTTCCTGAGAAAGCGAGC	58		
11F	GCTTCAGATGTTTGTCGGGC	58	188	ORF4 anti-repressor in phage vB_EcoP_ZX5
11R	ATAGTGCCTGGTGTCACTGC	58		
12F	GTCAGCGGACTGGCTAACTT	58	172	

12R	AATTCCAGCCATCACCAGGG	58		ORF10 endolysin in phage vB_EcoP_ZX5
13F	GGCATAGCAATTCGCGTTGT	56	198	ORF9 holin in phage vB_EcoP_ZX5
13R	AATAACCGCAGCCAGCATGA	56		

Table S2: Open reading frames (ORFs) of phage vB_EcoP_ZX5

ORF	Start	Stop	Strand	Length (bp)	Start codon	Function
ORF1	133	2	-	132	ATG	hypothetical protein
ORF2	658	155	-	504	ATG	hypothetical protein
ORF3	956	630	-	327	ATG	hypothetical protein
ORF4	1773	1078	-	696	ATG	anti-repressor
ORF5	1892	1779	-	114	GTG	ash family protein
ORF6	2346	2089	-	258	ATG	hypothetical protein
ORF7	2542	5661	+	3120	ATG	tail fiber domain-containing protein
ORF8	5968	6372	+	405	ATG	membrane protein
ORF9	6359	6667	+	309	ATG	phage holin family protein
ORF10	6657	7286	+	630	ATG	endolysin
ORF11	7283	7765	+	483	ATG	DUF2514 domain-containing protein
ORF12	8083	9333	+	1251	GTG	site-specific integrase
ORF13	9531	9337	-	195	ATG	hypothetical protein
ORF14	10178	9528	-	651	ATG	Adenine DNA methyltransferase
ORF15	10470	10171	-	300	GTG	perC transcriptional activator
ORF16	10583	10467	-	117	ATG	hypothetical protein
ORF17	10828	10580	-	249	ATG	AlpA family phage regulatory protein
ORF18	11759	10878	-	882	ATG	recombinase RecT
ORF19	12577	11756	-	822	ATG	exodeoxyribonuclease VIII/RecE-like protein

ORF20	13028	12876	-	153	ATG	hypothetical protein
ORF21	13766	13182	-	585	ATG	repressor
ORF22	13921	14151	+	231	ATG	hypothetical protein
ORF23	14493	15323	+	831	ATG	primosomal protein I
ORF24	15295	16071	+	777	ATG	DNA replication protein
ORF25	16189	16533	+	345	ATG	DUF1064 domain-containing protein
ORF26	16595	17158	+	564	ATG	eae-like protein
ORF27	17155	17388	+	234	ATG	hypothetical protein
ORF28	17385	18047	+	663	ATG	hypothetical protein
ORF29	18049	18240	+	192	ATG	hypothetical protein
ORF30	18243	18857	+	615	ATG	EaA-like protein
ORF31	18854	19207	+	354	ATG	phage ninx
ORF32	19200	19538	+	339	ATG	hypothetical protein
ORF33	19660	20253	+	594	ATG	terminase small subunit
ORF34	20250	21725	+	1476	GTG	terminase large subunit
ORF35	22154	21816	-	339	ATG	hypothetical protein
ORF36	22176	22427	+	252	ATG	hypothetical protein
ORF37	22861	23067	+	207	ATG	hypothetical protein
ORF38	23082	24761	+	1680	ATG	phage collar
ORF39	24758	25054	+	297	ATG	hypothetical protein
ORF40	25057	25752	+	696	ATG	peptidase
ORF41	25767	26753	+	987	ATG	capsid protein
ORF42	26805	27242	+	438	ATG	hypothetical protein
ORF43	27253	27582	+	330	ATG	hypothetical protein
ORF44	27633	27956	+	324	ATG	hypothetical protein
ORF45	27956	28561	+	606	ATG	hypothetical protein
ORF46	28561	31032	+	2472	ATG	hypothetical protein
ORF47	31032	31496	+	465	ATG	hypothetical protein
ORF48	31496	32041	+	546	ATG	hypothetical protein

ORF49	32041	34554	+	2514	ATG	virion protein
ORF50	34551	36353	+	1803	ATG	hypothetical protein
ORF51	36359	38833	+	2475	ATG	hypothetical protein
ORF52	39053	39433	+	381	ATG	hypothetical protein
ORF53	39563	39465	-	99	ATG	hypothetical protein
